# A novel in situ bone elevation method to achieve vertical periodontal augmentation in dogs: A pilot study

**DOI:** 10.1111/joor.12800

**Published:** 2019-05-09

**Authors:** Lili Li, Wenqi Su, Xiaoting Xie, Lang Lei, Jun Bao, Shasha He, Sheng Chen, Yan Yang, Fuhua Yan, Houxuan Li

**Affiliations:** ^1^ Department of Periodontology Nangjing Stomatological Hospital, Medical School of Nanjing University Nanjing China; ^2^ Central laboratory of Stomatology Nangjing Stomatological Hospital, Medical School of Nanjing University Nanjing China; ^3^ Department of Orthodontics Nangjing Stomatological Hospital, Medical School of Nanjing University Nanjing China; ^4^ Department of Pathology Nangjing Stomatological Hospital, Medical School of Nanjing University Nanjing China

**Keywords:** autogenous bone graft, bone augmentation, bone blocks, horizontal bone defects, periodontitis, regenerative medicine

## Abstract

**Objectives:**

The purpose of this study was to investigate whether a novel in situ interdental bone elevation method could achieve vertical bone augmentation around natural teeth.

**Methods:**

Horizontal periodontal bone defects were created at nine quadrants of mandibles in five dogs. Six weeks later, one of the nine quadrants was randomly chosen as the model control. The remaining mandibles were allocated into two experimental groups: cortical bone removing (CBR) or interdental bone elevation (IBE). For the IBE group, four millimetres of interdental bone blocks were separated and elevated from the base of alveolar bone. Then bone xenografts were implanted beneath the elevated alveolar blocks. Animals were euthanised 12 weeks post‐operation. Cone beam computed tomography (CBCT) examination and histological analysis were performed to evaluate the surgical outcomes.

**Results:**

Enhanced soft tissue profiles were observed in the two experimental groups as compared to the model control group. CBCT images showed that the height of alveolar bone was significantly higher in the IBE group with bone blocks seated near the cementoenamel junction. Significantly larger area of bone tissues with the highest coronal level of new bone was observed in the IBE group. New bone was observed around the elevated bone blocks with bone remodelling and neovascularisation inside the elevated blocks.

**Conclusions:**

Vertical bone augmentation at interdental sites may be performed through in situ interdental bone elevation for patients with horizontal alveolar bone resorption.

## 1 BACKGROUND

Periodontal disease is one of the most prevalent chronic inflammatory diseases worldwide and is the leading cause of tooth loss in adults.^1^, ^2^ Globally, about 5‐20% adults suffer from severe periodontitis, which leads to a compromised quality of life in the elderly.^3^

Periodontal tissue consists of gingiva, cementum, periodontal ligament and alveolar bone. With insertion of Sharpey's fibres into the adjacent cementum and alveolar bone, periodontal ligament anchors the tooth in the socket. Once lost, this delicate structure is difficult to regenerate. Investigators have struggled for years to regenerate the damaged periodontal tissues caused by periodontitis. Despite a predictable outcome of periodontal regenerative therapy in intra‐bony defects^4^, ^5^ and class II furcation involvement,^6^ the prognosis for horizontal alveolar bone resorption, the most common type of bone loss in periodontitis, remains poor.

Several factors contribute to the limited regenerative potential of horizontal bone defects: (a) difficulty in maintaining a stable three‐dimensional space for blood clot and regenerative materials; (b) lack of sufficient precursor cells/stem cells in the surgical area; (c) the harsh inflammatory microenvironment with microbial challenges and insufficient blood supply; (d) the delicate anatomical structure between adjacent teeth, which limits surgical procedures. Despite the poor prognosis for horizontal resorption in the periodontal region, extensive clinical and experimental evidence has demonstrated the successful use of guided bone regeneration to regenerate missing bone at implant sites with insufficient bone volume and the long‐term success of implants placed simultaneously with, or after, guided bone regeneration.^7^, ^8^ Urban et al^9^, ^10^, ^11^ showed that vertical and horizontal alveolar ridge augmentation in edentulous region can be achieved by mixed bone grafts (autograft:xenograft = 1:1) through “tent technique” or “sausage technique.”

Bone has the inherent ability to completely regenerate if it is provided with a fracture space or an undisturbed enclosed scaffold.^12^ Inspired by the concept of GBR in the edentulous alveolar ridge, Chopra et al^13^ reported a case with augmentation of bone in the interdental area with horizontal bone loss by building a contained defect using calcium phosphate cement to simulate the lost cortical plates. Such promising results indicated the feasibility of periodontal bone regeneration in the horizontal bone loss sites. However, this technique still faces the problem of lack of source of stem cells and adequate blood supply.

In this study, we designed a novel interdental bone elevation (IBE) technique to simulate the autologous bone transplantation, form bony walls to contain bone graft material, and generate a periodontal microenvironment to facilitate periodontal regeneration. In addition, a space‐maintaining PLA synthetic membrane further provides an enclosed space for stable clot formation and tissue regeneration. Our present pilot animal study demonstrated that the IBE technique may enhance periodontal bone regeneration in the horizontal periodontal bone loss model.

## 2 MATERIALS AND METHODS

The research protocol was approved by the Ethical Committee of Nanjing Stomatological Hospital, Medical School of Nanjing University. Five male 1‐year‐old healthy beagle dogs, weighing approximately 10 kg, were used in this study. The animals were raised in kennels in the Laboratory Animal Centre of Nanjing Drum Tower Hospital. They were fed with moistened balanced canine food and had free access to water. The experimental schedule is shown in Figure S1.

One of the dogs was randomly chosen as a control. Horizontal periodontal bone defects were created at the right mandible of the control dog. The left mandible of the control dog remained untouched. As for the other four dogs, horizontal periodontal bone defects were created at left and right mandibles. At the time of regenerative surgery, two mandibles of each dog were randomly assigned to the cortical bone removing (CBR) group or the interdental bone elevation (IBE) group. Regenerative surgeries were done in the interdental sites from the second premolar to the first molar. Thus, each dog carried three sites of the CBR method and three sites of the IBE method.

### 2.1 Establishment of periodontal bone defect model

Animals were routinely fasted overnight before the operation. They were given an intramuscular injection of atropine (0.025 mg/kg) 10‐15 minutes prior to anaesthesia induction using xylazine hydrochloride (0.1 ml/kg, Shengda Animal Drug Co., Ltd). When basic anaesthesia was achieved, propofol (Xi'an Li Bang Pharmaceutical Co., Ltd) was intravenously injected to maintain anaesthesia. After skin preparation and draping were completed, supragingival scaling was performed using ultrasonic equipment. Then crevicular incisions were made and full‐thickness flaps were elevated from the first premolar to the first molar of mandible on both buccal and lingual sides. The interdental alveolar bone was removed 4 mm away from the cementoenamel junction (CEJ). Markers were made at the corresponding root surface. The exposed periodontal ligament was scaled by manual curettes. Finally, flaps were repositioned and properly sutured (Figure S2). Penicillin sodium (800 000 U) was intramuscularly injected daily post‐operation for 3 days.

To simulate alveolar bone loss caused by periodontitis, sufficient time was given to induce root infection and gingival inflammation. After 6 weeks of plaque accumulation, the alveolar bone defect model with soft tissue inflammation was successfully established.

### 2.2 Periodontal regenerative surgery

Firstly, periodontal basic treatment was performed with ultrasonic instrument to remove tartars and biofilms from the tooth surface. Then crevicular and vertical incisions were made between the mesial angle of the first premolar and the distal angle of the first molar at the buccal side. Crevicular incision was also made at the lingual side. The mucoperiosteal flap was elevated. Thorough debridement was performed using manual and ultrasonic instruments. The teeth surfaces were treated with minocycline hydrochloride (Sunstar, Japan) prior to grafting.

For the CBR group, the cortical bone of the alveolar ridge was removed. Then a preformed PLA membrane (Guidor, Sunstar, Japan) was positioned onto the lingual bone margin. Thereafter, xenograft bone graft (Bio‐Oss, Geistlich) was placed on the recipient site, and then covered and shaped by the PLA membrane.

**Figure 1:**
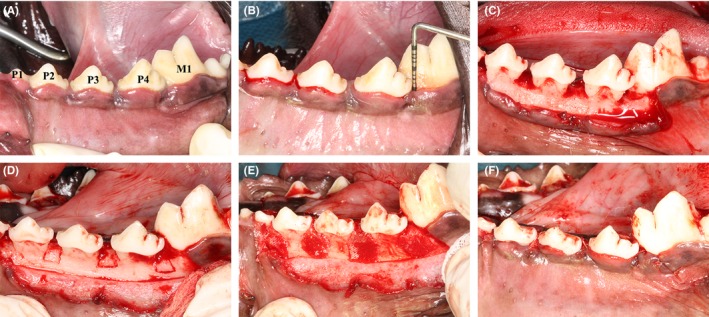
Clinical photographs demonstrating the operation steps of the in situ interdental bone elevation method for vertical periodontal regeneration. (A) Six weeks after modelling, obvious gingival collapse in the proximal sites and gingival swelling were observed with calculus accumulated. (B) Periodontal probing was performed after supragingival scaling. The probing depth was measured to be 2.29 ± 0.43 mm in the proximal sites. (C) The interdental bone defects after 6 wk of modelling. (D) The bone incisions were located using a round bur. (E) The autologous bone block was elevated by 4 mm and bone graft (Bio‐Oss, Geistlich) was placed beneath it. The regenerative space was covered and shaped by a preformed PLA membrane. (F) The flaps were repositioned and properly sutured. Multiple periosteum incisions were performed to ensure that the wound could be closed without tension [Colour figure can be viewed at http://wileyonlinelibrary.com]

For the IBE group, the bone incision was made 4 mm below the alveolar bone ridge and 0.5‐1 mm to the tooth surface (Figure 1). The osteotomy was performed using a fine diamond dental bur while cooling with large irrigation of cold saline. Then the autologous bone block was elevated by 4 mm and bone graft (Bio‐Oss, Geistlich) was placed beneath it. Thereafter, the regenerative space was covered and shaped by a preformed PLA membrane.

Before wound closure, multiple periosteum incisions were performed so that the flaps could be fully released and the wound could be closed without tension. Finally, horizontal mattress sutures and interrupted sutures were applied to ensure wound fixation and adaptation. Penicillin sodium (800 000 U) was intramuscularly injected daily post‐operation for 3 days. The surgical sites were examined at the second, 6th and 12th week post‐operation.

### 2.3 Radiographic examination

After euthanasia and sampling, radiographic images of defect sites were obtained with a cone beam CT apparatus NewTom VG (NewTom Co., Ltd., http://www.newtom.it). Scanning parameters were set as 110 kV, 32‐38 MAS, 360 degrees rotation, high‐resolution zoom, 3.6 seconds scanning and 0.125 mm image layer thickness. The original volume was calculated to obtain axial images. Three‐dimensional reconstructions were performed using a software NNT viewer (version 5.3), in which optimal visualisation of the images was obtained through image‐processing tools.

### 2.4 Histological processing

Twelve weeks post‐surgery, the animals were anesthetised and euthanised by an overdose of propofol (Xi'an Li Bang Pharmaceutical Co., Ltd). Formaldehyde solution was poured into bilateral carotid artery. Six specimens of each dog were harvested and fixed in 4% phosphate‐buffered paraformaldehyde phosphate for 3 days. Then the specimens were immersed in a decalcifying solution containing EDTA (G1105, Servicebio). Decalcifying solution was changed every 3 days until complete demineralisation. The specimens were cut in a mesio‐distal direction. Sections (5 μm in thickness) were prepared and stained with haematoxylin and eosin (HE) or Masson Trichrome. Sections were scanned by NanoZoomer S60 (Hamamatsu Photonics Co., Ltd). Micrographs were observed and analysed using Caseviewer (3DHISTECH Ltd).

The following parameters were measured using the Image‐Pro Plus 6.0 (Media Cybernetics) as previously described^14^, ^15^ (a) the length of newly formed periodontal ligament (LNP), defined as the distance from the notch on the root to the coronal border of regenerated periodontal ligament; (b) the length of newly formed cementum (LNC), defined as the distance from the notch on the root to the coronal border of regenerated cementum; (c) the length of junctional epithelium (LJE); (d) the height of new bone (Notch‐C), defined from the notch on the root to the coronal extension of new bone; (e) the height of the bulk tissues (Notch‐Cbt), defined from the notch on the root to the coronal extension of the bulk tissues; (f) the length of the connective tissue (CT), defined as the distance from the apical endpoint of junctional epithelium to Cbt.

### 2.5 Statistical analysis

Data were statistically analysed using GraphPad Prism version 5.0 (GraphPad Software Inc). All results were presented as mean ± standard deviation (SD). Kolmogorov–Smirnov test was applied to examine the data normality. Significant differences in mean values among the groups were determined by one‐way ANOVA. Bartlett's test was employed for equal variances. Student–Newman–Keuls test was utilised for multiple comparisons. *P* < 0.05 was considered as statistical significance.

## 3 RESULTS

### 3.1 Parameters of periodontal bone defect model

Clinical periodontal examinations were conducted before regenerative surgery to ensure the establishment of periodontal bone defect model. Six weeks after model establishment, gingival recessions were observed in some sites and probing depth in the proximal surgical sites were 2.29 ± 0.43 mm, which was significantly deeper than the blank control. Calculus accumulation and gingival inflammation were also observed (Figure 1). The bone defect dimensions are shown in Table 1.

**Table 1:** The bone defect dimensions of the periodontal bone defect model at the time of regenerative surgery (mean values ± standard deviations; mm)

Site	Buccal‐lingual (n = 9)	Vertical (n = 9)	Mesial‐distal (n = 9)
2nd premolar	5.13 ± 0.85	4.6 ± 0.89	4.7 ± 0.84
3rd premolar	4.75 ± 0.50	4.7 ± 0.67	4.0 ± 0.61
4th premolar	6.0 ± 1.15	4.5 ± 0.99	4.6 ± 1.39

### 3.2 Wound healing after regenerative surgery

**Figure 2:**
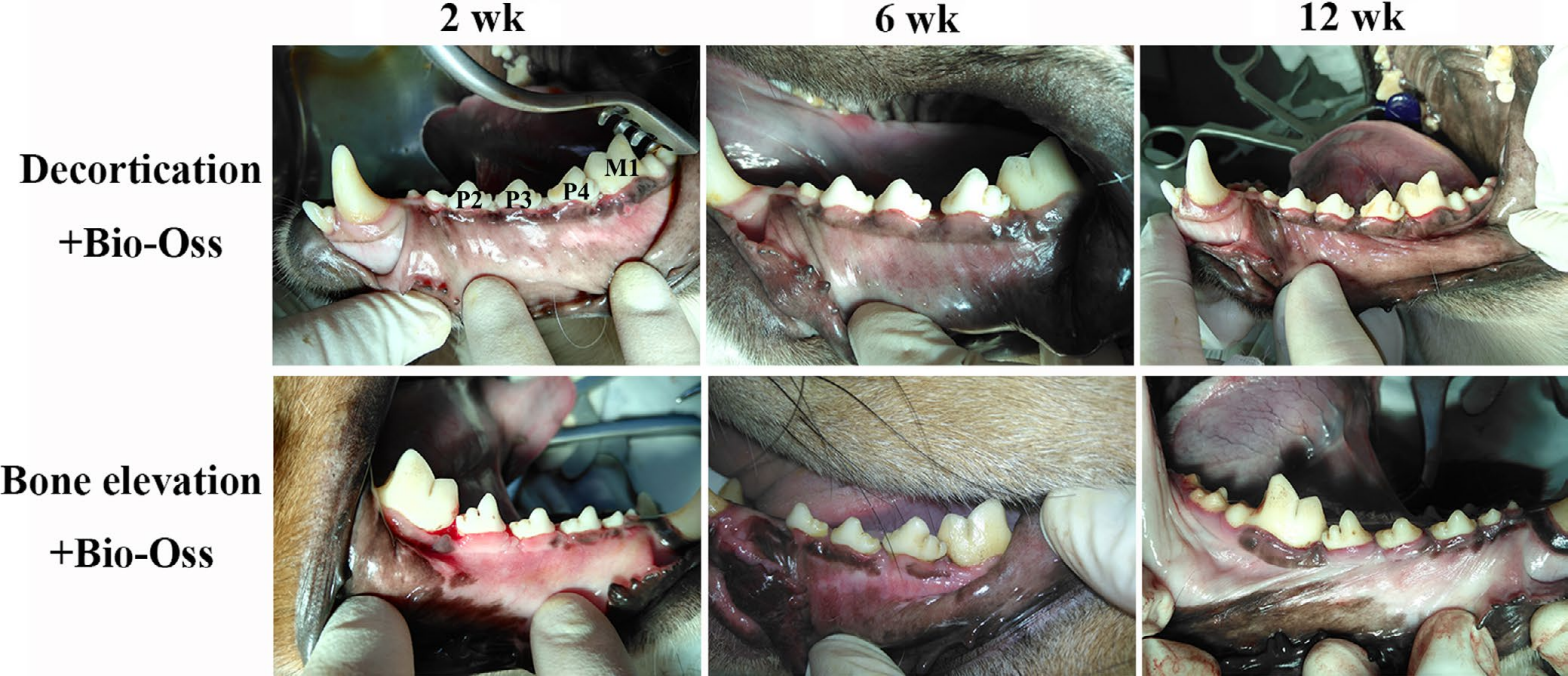
Clinical photographs showing the wound healing at the 2nd, 6th and 12th wk after surgery. Membrane exposure was observed at the 2nd wk post‐surgery. Minocycline ointment was utilised locally to avoid infections. The wounds healed well without conspicuous infection at the 6th wk post‐operation. Surgical sites obtained complete soft tissue coverage at the 12th wk [Colour figure can be viewed at http://wileyonlinelibrary.com]

Two weeks after periodontal regenerative surgery, about 2‐3 mm membrane exposure occurred at some sites in the CBR and IBE groups. Those sites were treated with minocycline hydrochloride ointment under close observation. The wound healed with no further adverse conditions. At the 6th week post‐operation, no conspicuous infection was found and the wounds had healed in all groups. At the 12th week, the soft tissue showed complete closure (Figure 2).

Collapse of soft tissue and loss of gingival papilla were observed in the bone defect group without any graft. Surgical sites of the CBR and IBE groups showed good soft tissue profile, without unacceptable recession.

### 3.3 Periodontal regeneration at surgical sites

**Figure 3:**
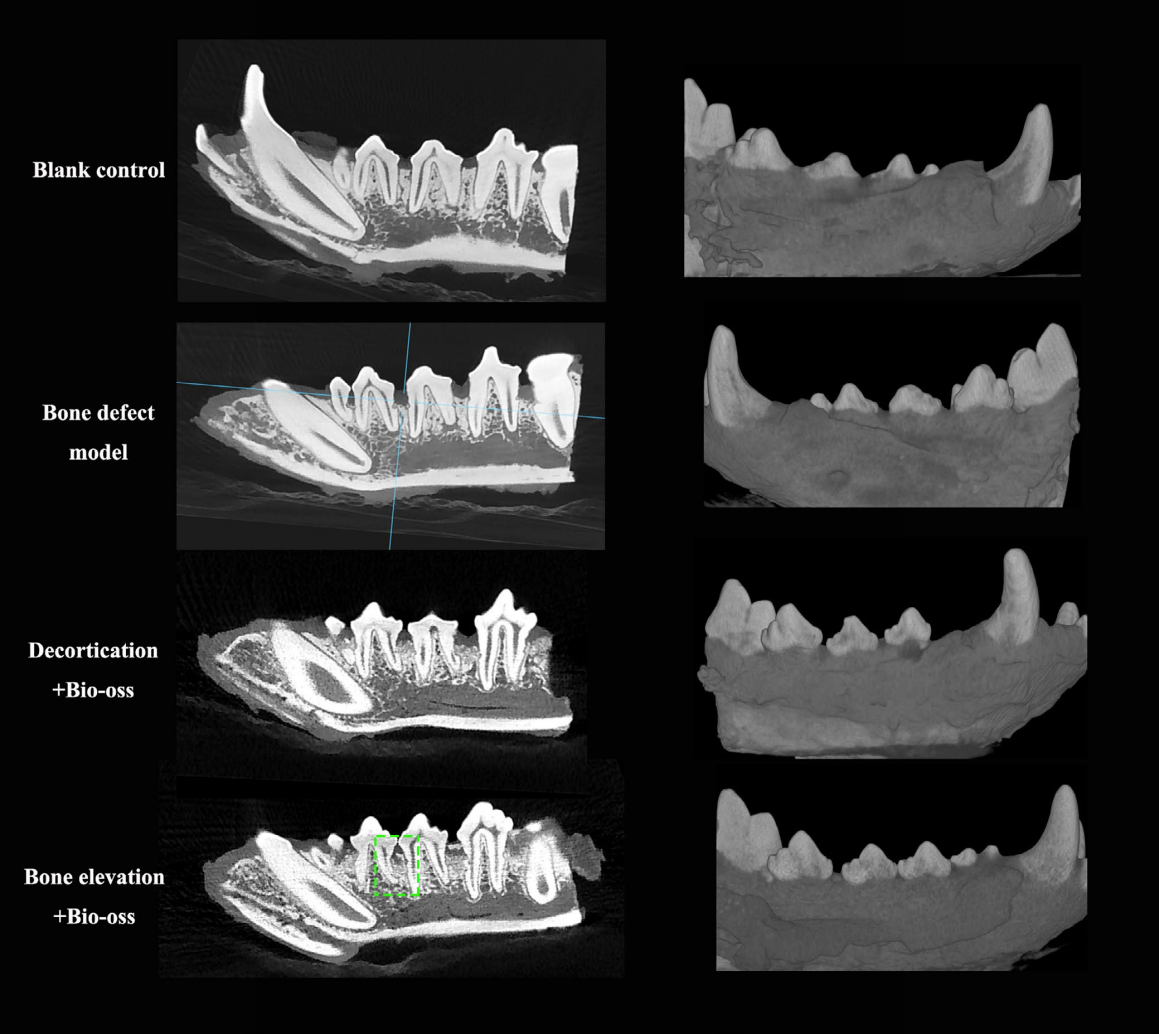
Cone beam CT scans of specimens. Images of the CBR group indicate that parts of the grafted materials had been lost since the volume of the bulk tissue decreased greatly. In the IBE group, images of elevated bone blocks and grafting materials were in situ. The elevated bone blocks were still seated near the CEJ level. The green box highlights a site with bone integration between the elevated bone block and recipient bone bed [Colour figure can be viewed at http://wileyonlinelibrary.com]

To evaluate the outcome of periodontal regenerative surgeries, cone beam CT images were analysed (Figure 3). In the CBR group, images of grafted materials were observed at the interdental sites. However, part of the grafted materials had been lost since the volume of the bulk tissue decreased significantly. In the IBE group, images of elevated bone blocks and grafting materials were observed. The bone blocks were seated near the CEJ level. Integration between the elevated bone block and the recipient bed was observed at some sites.

**Figure 4:**
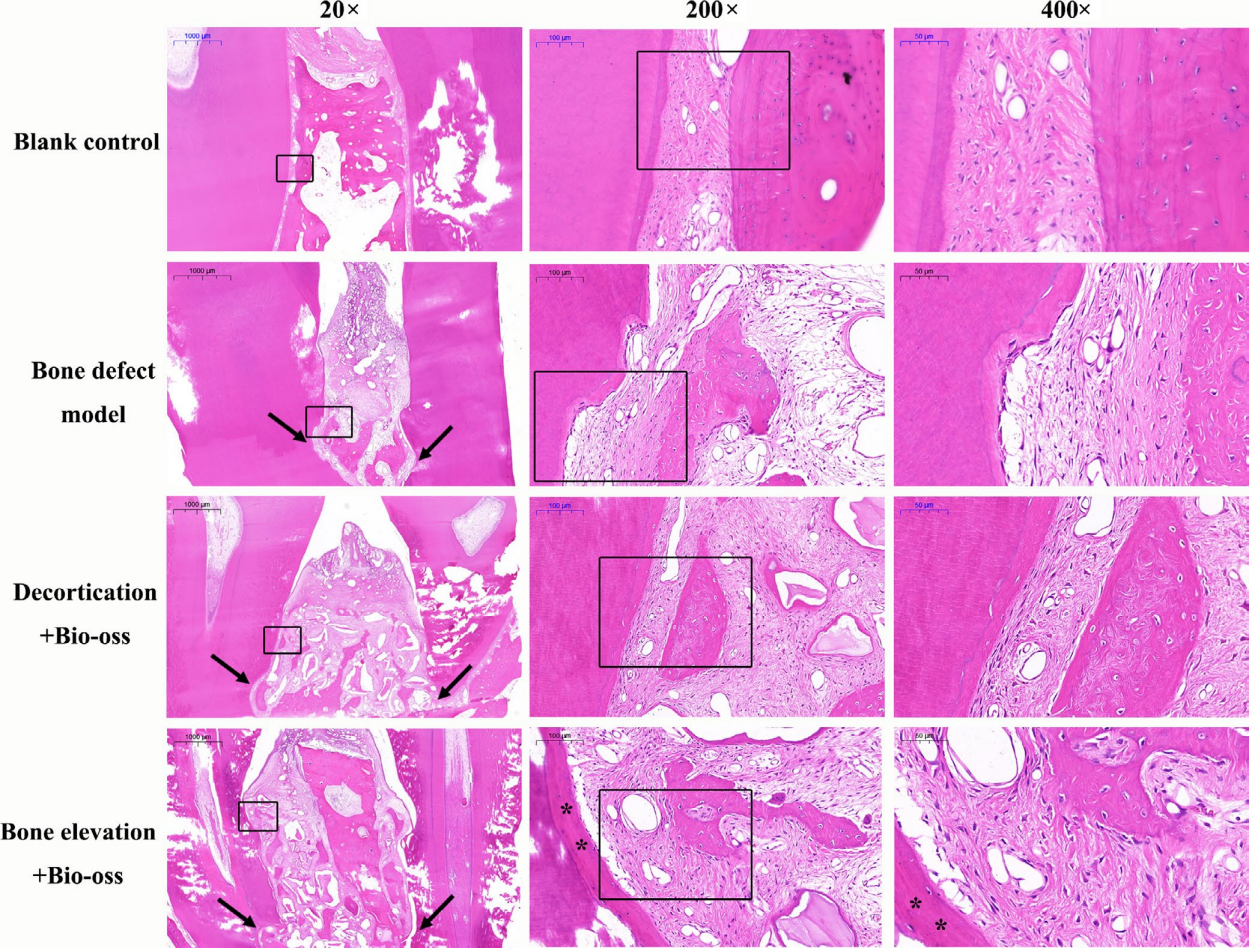
Mesio‐distal sections of the surgery sites stained with HE. The black arrows indicate the notch as markers to present bone level before periodontal regenerative surgery. The black stars indicate the newly formed cementum‐like tissue on the root surface. Regions in the boxes are shown next in a greater magnification [Colour figure can be viewed at http://wileyonlinelibrary.com]

**Figure 5:**
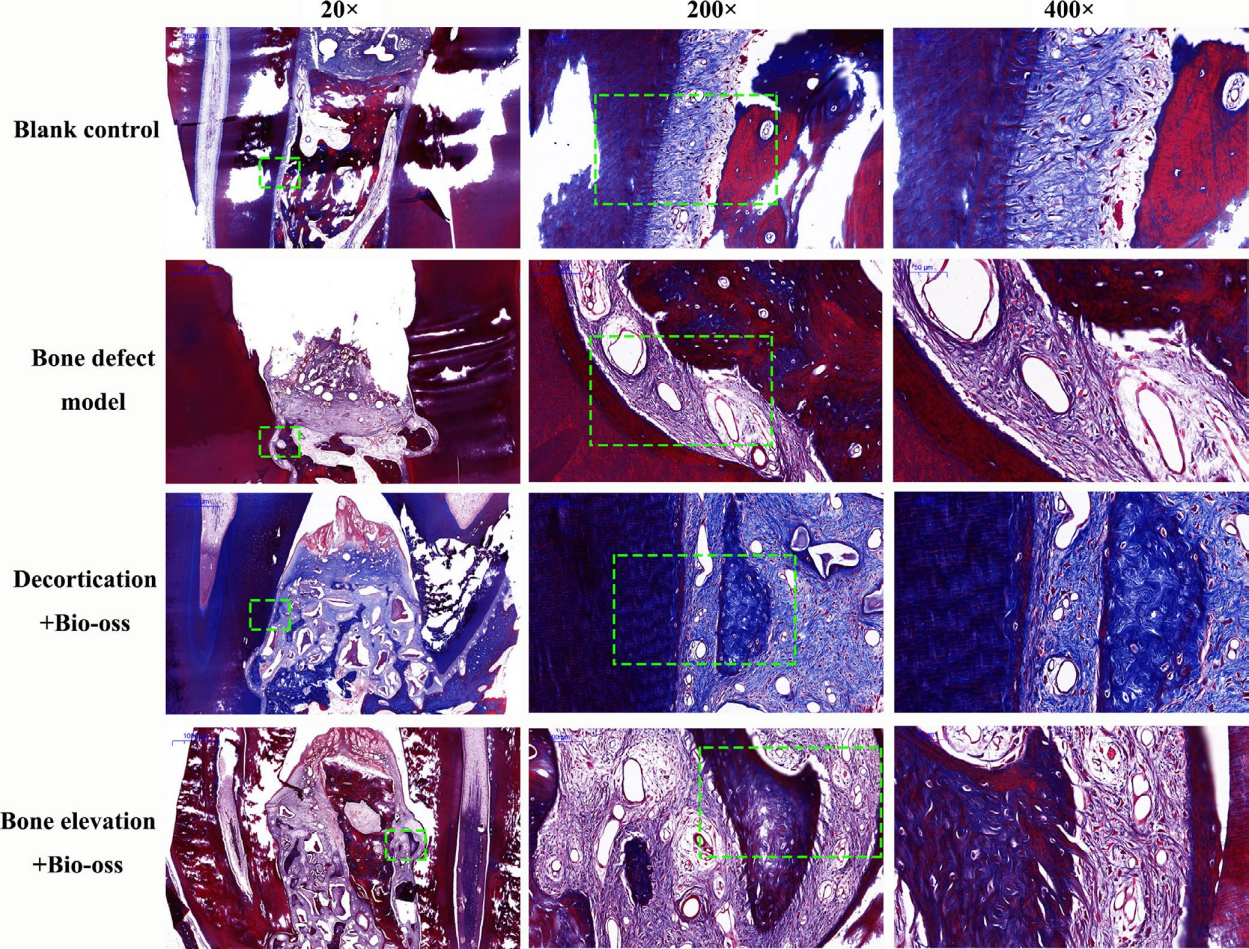
Mesio‐distal sections of the surgery sites stained with Masson trichrome reagents. Newly formed periodontal ligament could be observed clearly. Although it is still not as orderly as the fibre bundles in natural periodontal ligament, the periodontal gap is distinct. Regions in the boxes are shown next in a greater magnification [Colour figure can be viewed at http://wileyonlinelibrary.com]

Histological analysis was performed in HE and Masson's trichrome‐stained pathological sections. The surgical sites in the model control group showed a small amount of bone regeneration within the notch on the roots. This group healed with long connective tissue or severe gingival recession (Figures 4 and 5). Better outcomes were found in the two experimental groups. Significant differences were observed among the groups in terms of the length of connective tissue, distance from the notch to the coronal endpoint of the newly formed bone, as well as the distance from the notch to the coronal endpoint of the bulk tissues (Table 2). As compared to the model control and CBR groups, surgical sites of the IBE group had significantly larger area of bulk tissue and higher coronal level of newly formed bone, which indicated more space for tissue regeneration and maturation. Interestingly, new cementum‐like tissue with cells formed on the root in all groups, as well as the newly formed periodontal ligament. Although it was not as orderly as the fibre bundles in natural periodontal ligament, the periodontal gap was distinct. Few areas of ankylosis were observed (Figure 4).

**Table 2:** Results of histological measurements (mean values ± standard deviations)

Parameters	Model control (n = 6)	Decortication (n = 16)	Bone elevation (n = 18)
LNP (mm)	1.43 ± 0.44	2.11 ± 0.59^a^	2.94 ± 0.76^a^, ^b^
LNC (mm)	1.11 ± 0.40	2.00 ± 0.80^a^	2.73 ± 1.07^a^
LJE (mm)	0.50 ± 0.22	0.63 ± 0.23	0.71 ± 0.34
CT (mm)	1.52 ± 0.74	0.24 ± 0.10^a^	0.24 ± 0.09^a^
N‐C (mm)	0.83 ± 0.26	2.10 ± 0.39^a^	3.23 ± 0.55^a^, ^b^
N‐Cbt (mm)	0.82 ± 0.24	2.17 ± 0.44^a^	3.73 ± 0.985^a^, ^b^
ABT (mm^2^)	2.68 ± 0.85	6.66 ± 1.31^a^	11.27 ± 1.66^a^, ^b^

Abbreviations: ABT, area of bulk tissue; CT, length of connective tissue; LJE, length of the newly formed junctional epithelium; LNC, length of the newly formed cementum‐like tissue; LNP, length of the newly formed periodontal ligament; N‐C, distance from the notch on the root to the top of the newly formed bone; N‐Cbt, distance from the notch to the top of the most coronal peak of the bulk tissue (Cbt).

a Statistically significant difference compared with model control group (*P* < 0.05).

b Statistically significant difference compared with decortication group (*P* < 0.05).

### 3.4 Reconstruction of the elevated bone blocks

**Figure 6:**
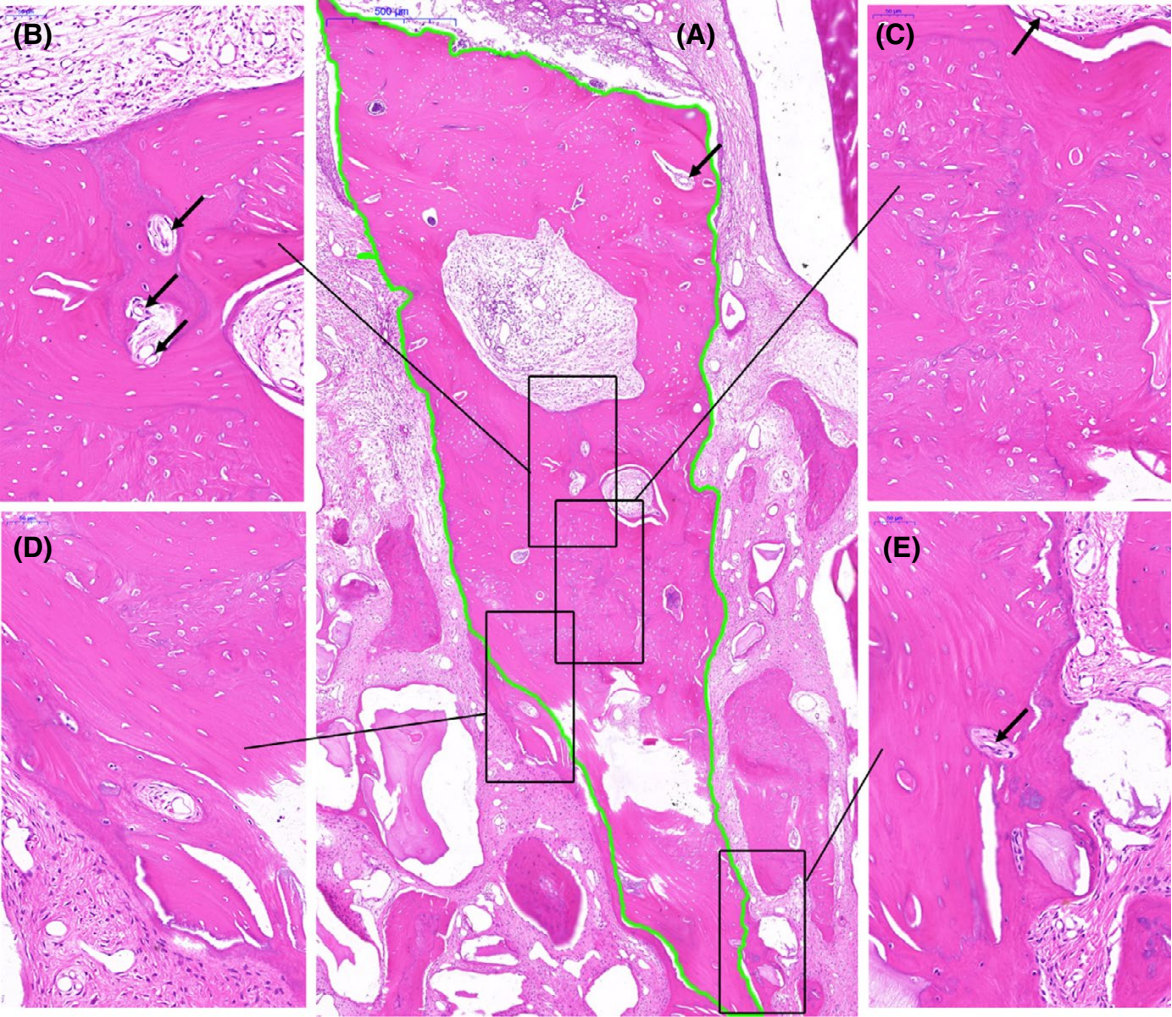
Reconstruction of the elevated bone blocks. (A) The overall view and boundary of the elevated bone block. (B, C) Newly formed bone inside the bone block. (D, E) The interface between the elevated autogenous bone and new bone. Black arrow indicates vessels growing into the elevated bone block [Colour figure can be viewed at http://wileyonlinelibrary.com]

In the IBE group, some bone blocks were lost due to inadequate fixation and excessive packing during placement. Those blocks were exposed within 2 weeks post‐operation and removed to ensure wound healing. At sites where the elevated bone block remained in situ, the blocks were surrounded by newly formed bone and directly in contact with the recipient bone. Neovascularisation was observed inside the elevated free bone blocks (Figure 6). Although there were areas with empty osteocyte lacunae and no normal vessels in the harversian canals, bone rebuilding and creeping substitution can be observed both inside and outside the bone blocks. The area of viable bone inside the autografts increased with the decrease in the distance to the resident bed (Figure 6).

## 4 DISCUSSION

Regeneration of periodontal horizontal bone loss is a major challenge for periodontists. This novel in situ interdental bone elevation method demonstrated the feasibility to regenerate the horizontal periodontal bone defects around natural teeth. At the IBE sites, the elevated bone blocks went through creeping substitution. Newly formed bone with vital osteocytes was found in the elevated bone blocks with blood vessels growing into the elevated alveolar bone indicating that the blocks may accomplish reconstruction.

Due to lack of the three‐dimensional space for holding regenerative materials and even stem cells, treatment outcome of horizontal alveolar bone resorption is less favourable as compared to the intra‐bony defect. Successful periodontal regeneration was reported in the furcation area around natural teeth in beagle dogs in a critical size horizontal bone loss model^16^ However, it must be noted that the anatomical features are different in the furcation area as compared to the interdental area. The “enclosed” cementum and alveolar bone‐walled furcation defect provides a regenerative niche of profuse source of stem cells and differentiation clues by the surrounding structures. In addition, the secluded nature of furcation area favours the regeneration process with a less contaminated and inflammatory microenvironment.

Advances in guided bone regeneration in edentulous region offer new opportunities for periodontal regeneration therapy. Several methods have been developed to regenerate bone including block bone graft, guided bone regeneration, distraction osteogenesis, “tent technique” and “sausage technique”^9^, ^17^, ^18^ Studies have confirmed the reliability of alveolar ridge augmentation in edentulous region. However, the horizontal bone resorption around natural teeth has long been a clinical due to the delicate size of the defect.

Inspired by cortical bone removal technique and onlay bone graft, we tried these two methods around natural teeth. In our IBE method, the elevation of alveolar process from the basal bone formed a three‐dimensional space with two decorticalised bone walls in the coronoapical direction and two cementum walls in the mesio‐distal direction, which favored periodontal tissue regeneration. Indeed, the present preliminary study demonstrated that IBE could possibly enhance periodontal regeneration.

A major issue of the IBE method is the viability and survival of the elevated alveolar bone. The histological reconstruction process of the elevated bone blocks is similar to that of the autogenous bone blocks in GBR in implant dentistry. Autologous bone block grafting is a reliable method for alveolar bone augmentation^19^ Rocchietta et al^20^ performed a clinical and histological study in humans on vertical bone augmentation with an autogenous block or particles. After 6‐10 months of healing, the block grafts showed satisfactory bone filling, revascularisation and bone remodelling. In another clinical study of anterior maxilla augmentation using palatal bone block, all but one bone blocks integrated successfully after 4 months^21^ In addition, animal studies showed that autologous transplantation of bone blocks can achieve 77 ± 6.2% of vital bone in beagle dogs after 6 months healing period^22^ Although partial necrotic areas were observed in two samples at week 4, bone regeneration and creeping substitution continued in a tibia transplantation model in rabbits, and trabecular connection with recipient bone was observed at week 12^23^ Therefore, appearance of necrotic bone in some EIB sites in our model does not imply failure. Histological analysis revealed new bone formation and revascularisation inside the necrotic bone, which indicated the ongoing creeping substitution. Hence, it is reasonable to predict that the necrotic parts of the elevated alveolar bone blocks will be substituted through creeping substitution.

Despite favourable results in the elevated blocks, drawbacks in the present method must be noted. Three of the 12 elevated bone locks were expelled from surgical sites. Such failure may arise from soft tissue dehiscence, a common problem in vertical or horizontal GBR^24^ Although multiple periosteum incisions were utilised to release tension of the flaps, primary closure of the surgical area remained challenging to ensure undisturbed bone regeneration. Moreover, blood supply of the interdental soft tissue only comes from the base, which makes it highly fragile and requires more careful management during periodontal regenerative surgery^25^ Furthermore, self‐comparison of alveolar bone before and after IBE by CBCT may provide more information on the outcome of periodontal regeneration.

In conclusion, although regeneration of horizontal bone loss around natural tooth is a great challenge, the present study suggests that the IBE method may bring hope to overcome this predicament. And advances in microscopic dental instruments may further facilitate the surgical process for handling small bone blocks. However, since the sample size is limited, further investigations are needed to expand sample size and draw a more precise and predictable conclusion. Furthermore, longer observation is needed to confirm the long‐term prognosis of this method.

## CONFLICT OF INTEREST

All the authors declare no conflict of interest in connection with this article.
