# Supplementary material for: A novel in situ bone elevation method to achieve vertical periodontal augmentation in dogs: A pilot study
Source: J Oral Rehabil. 2019 May 9;46(8):756–64. doi: 10.1111/joor.12800 (PMC6852005; doi:10.1111/joor.12800)
Supplement: Supplementary file 1 [file JOOR-46-756-s001.pdf]

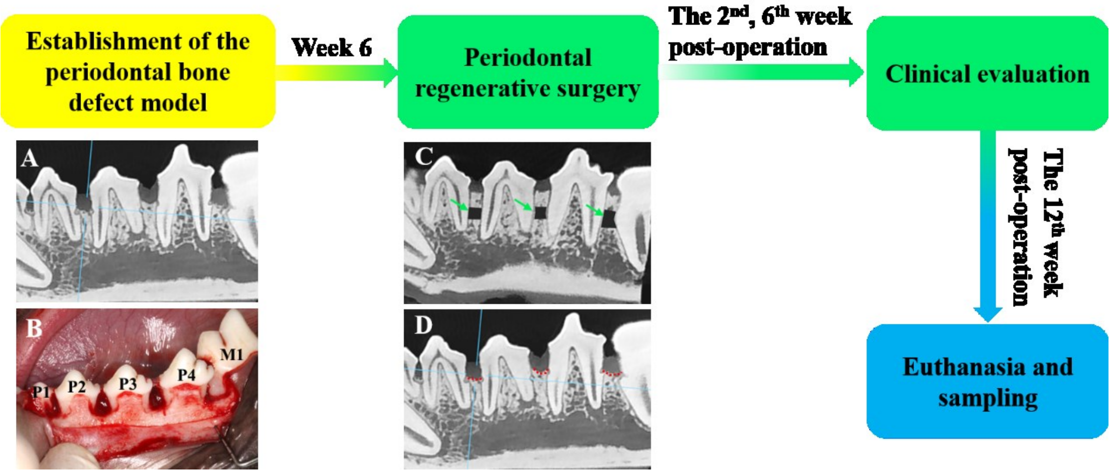

Supplement figure S1. Schematic drawing illustrating the arrangement of the experiment, as well as the periodontal bone defect model and two methods to study.

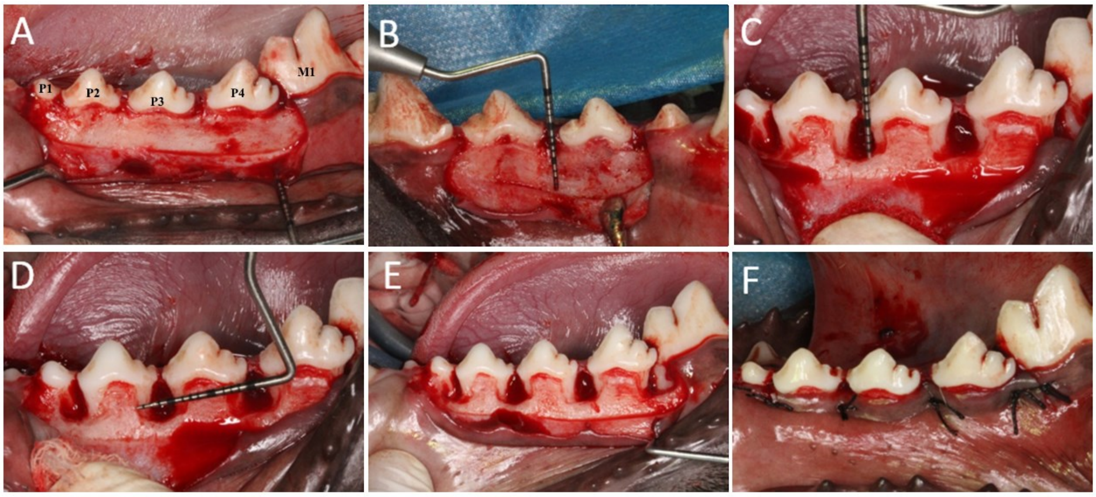

Supplement figure S2. Clinical photos presenting the model-building process of the periodontal bone defects. (A) Full-thickness flaps were elevated from the first premolar to the first molar of mandible on both buccal and lingual sides. (B-E) The interdental alveolar bone was removed 4 mm away from the cemento-enamel junction. (F) The flaps were repositioned and properly sutured

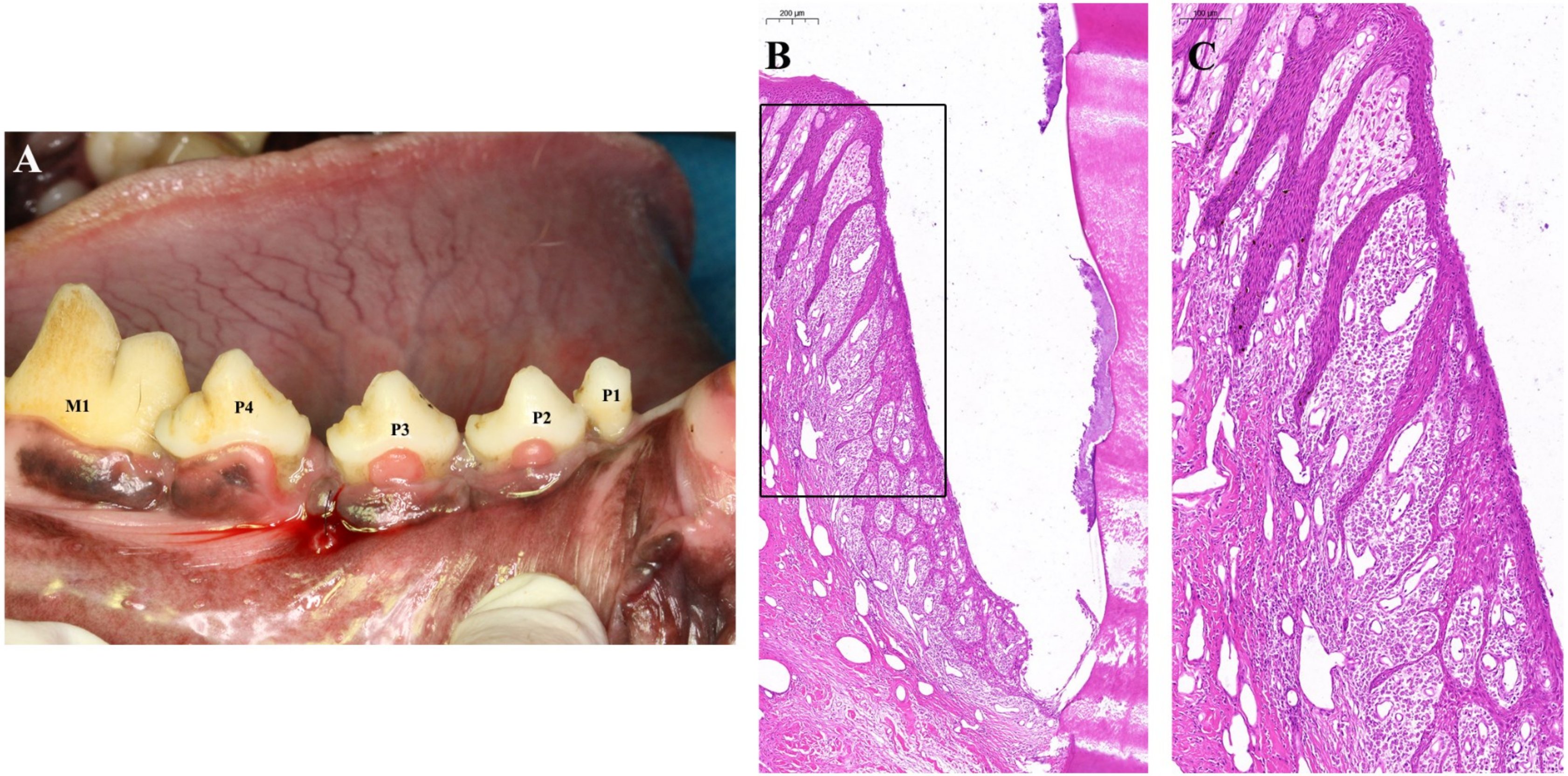

Supplement figure S3. The successful establishment of alveolar bone defect model with soft tissue inflammation. (Supplement figure S3A) Amounts of calculus accumulation, gingival inflammation and obvious gingival recessions were observed after six weeks of modeling. (Supplement figure S3B and S3C) Soft tissue inflammation could be seen in histopathological micrographs including elongation of epithelial spikes, inflammatory cell infiltration and connective tissue destruction.

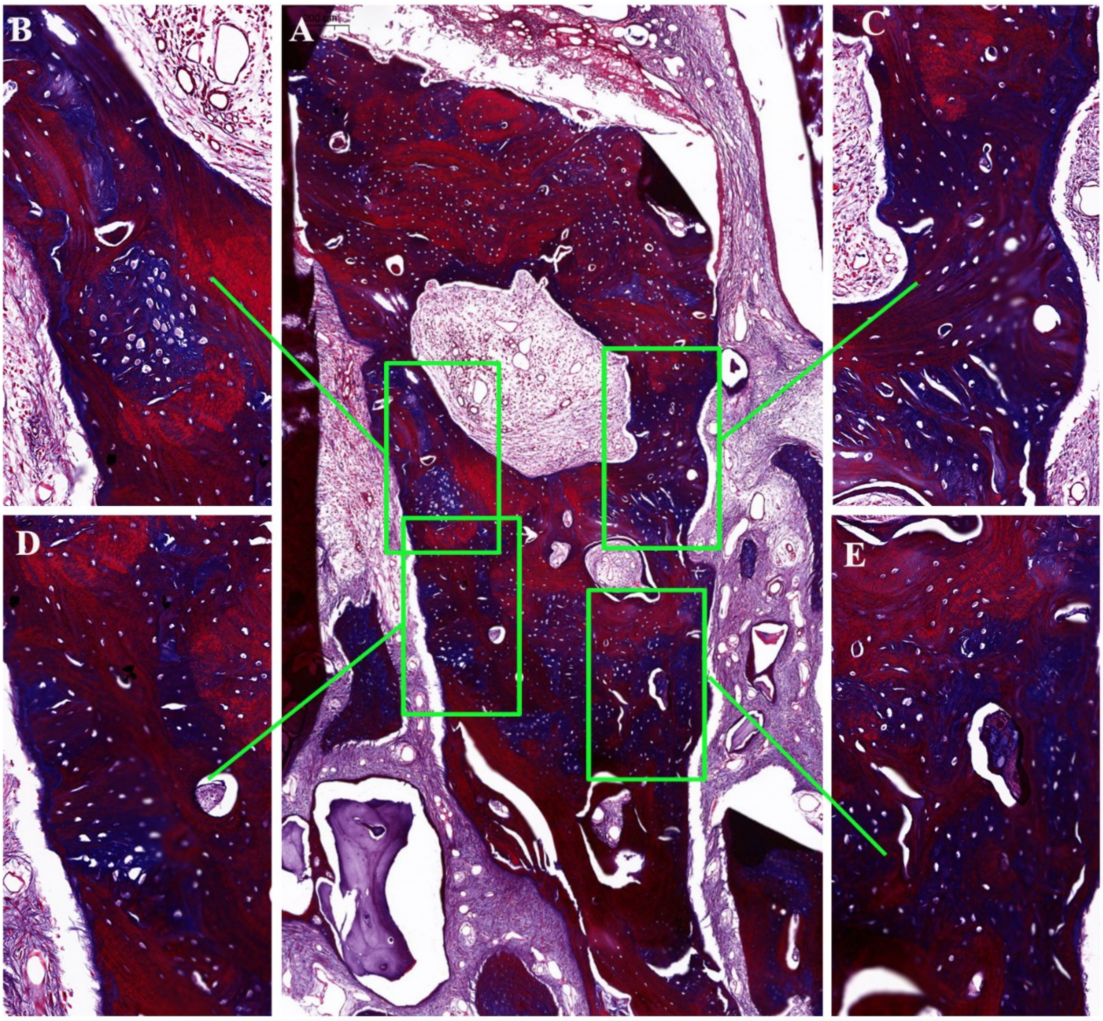

Supplement figure S4. Reconstruction of the elevated bone blocks by Masson Trichrome stain. (A) The overall view and boundary of the elevated bone block. (B, C, D, E) Newly formed bone inside the bone block.
